# Associations between Maternal Body Composition and Appetite Hormones and Macronutrients in Human Milk

**DOI:** 10.3390/nu9030252

**Published:** 2017-03-09

**Authors:** Sambavi Kugananthan, Zoya Gridneva, Ching T. Lai, Anna R. Hepworth, Peter J. Mark, Foteini Kakulas, Donna T. Geddes

**Affiliations:** 1 School of Human Sciences, The University of Western Australia, Crawley WA 6009, Australia; 21141062@student.uwa.edu.au (S.K.); peter.mark@uwa.edu.au (P.J.M.); 2 School of Molecular Sciences, The University of Western Australia, Crawley WA 6009, Australia; ching-tat.lai@uwa.edu.au (C.T.L.); anna.hepworth@uwa.edu.au (A.R.H.); foteini.kakulas@uwa.edu.au (F.K.); donna.geddes@uwa.edu.au (D.T.G.)

**Keywords:** leptin, adiponectin, maternal body composition, percentage fat mass, lactation, human milk, breastfeeding, appetite hormones, macronutrients, protein, lactose

## Abstract

Human milk (HM) appetite hormones and macronutrients may mediate satiety in breastfed infants. This study investigated associations between maternal adiposity and concentrations of HM leptin, adiponectin, protein and lactose, and whether these concentrations and the relationship between body mass index and percentage fat mass (%FM) in a breastfeeding population change over the first year of lactation. Lactating women (*n* = 59) provided milk samples (*n* = 283) at the 2nd, 5th, 9th and/or 12th month of lactation. Concentrations of leptin, adiponectin, total protein and lactose were measured. Maternal %FM was measured using bioimpedance spectroscopy. Higher maternal %FM was associated with higher leptin concentrations in both whole (0.006 ± 0.002 ng/mL, *p* = 0.008) and skim HM (0.005 ± 0.002 ng/mL, *p* = 0.007), and protein (0.16 ± 0.07 g/L, *p* = 0.028) concentrations. Adiponectin and lactose concentrations were not associated with %FM (0.01 ± 0.06 ng/mL, *p* = 0.81; 0.08 ± 0.11 g/L, *p* = 0.48, respectively). Whole milk concentrations of adiponectin and leptin did not differ significantly over the first year of lactation. These findings suggest that the level of maternal adiposity during lactation may influence the early appetite programming of breastfed infants by modulating concentrations of HM components.

## 1. Introduction

Human milk (HM) is the optimal nutrition for term infants as it contains a uniquely balanced profile of macronutrients along with micronutrients, hormones, antibodies, bioactive molecules [1] and cells [2,3], which adequately support the nutritional needs, appropriate growth, immunoprotection and physiological development of the infant [4,5]. It is well documented that prolonged breastfeeding is associated with decreased prevalence of overweight and obesity across the life course [6,7]. Enhanced appetite control in adults who have been breastfed as infants has been partly attributed to regulatory appetite hormones present in HM [8,9,10], which include leptin and adiponectin [8,11]. In rat pups, acute or chronic administration of leptin by intraperitoneal injection has been shown not to reduce food consumption but to modulate the expression of neuropeptides and receptors involved in the regulation of feeding behaviour (neuropeptide Y (NPY) and proopiomelanocortin (POMC)) [12], while in mice pups leptin deficiency caused profound disruptions in the development of the projections of the arcuate nucleus of the hypothalamus [13]. These findings indicate that leptin plays a neurotrophic role and contributes to the developmental programming of the hypothalamic appetite circuitry during the neonatal period, preceding leptin’s acute regulation of food intake in adults. Adiponectin has anti-inflammatory properties and improves both fatty acid metabolism and sensitivity to insulin [14]. In mice, adiponectin inhibits tension-sensitive gastric vagal afferent mechanosensitivity, modulating satiety signals in both lean and obese animals, while simultaneously increasing the mechanosensitivity of mucosal gastric vagal afferent in an obesity-induced model [15].

Additionally, the concentration of macronutrients in HM, namely protein, fat and lactose, may also be involved in regulation of the infant appetite control [9,16]. Differences in the concentrations of these factors may partly explain the variability in breastfeeding patterns observed in infants who feed on demand [17]. As such, an understanding of the factors that affect concentrations of appetite hormones and macronutrients in HM is critical, as it presents a unique opportunity for the prevention of unfavourable early developmental programming and subsequent obesity.

Leptin is secreted into the maternal circulation by white adipocytes and is subsequently transferred into the mammary ductal system via diffusion or a receptor-mediated transport mechanism [18]. Leptin is also contributed by lactocytes [19,20]. Previous studies have identified a positive correlation between maternal body mass index (BMI) and both maternal serum [21,22] and skim HM leptin [21,22,23,24], despite differing methodologies in leptin measurement [25]. However, no consistent relationship between maternal adiposity and HM leptin concentrations has been shown across lactation, with Miralles et al. (2006) reporting only moderate correlations between maternal BMI and HM leptin during the first 6 months of lactation (*R* = 0.387; *p* < 0.01) [8], while Bronsky et al. (2011) saw no consistent relationship over the first 12 months of lactation [11], despite utilizing a similar leptin measurement methodology. 

There are a number of factors that may contribute to these conflicting findings. BMI is a poor measure of adiposity, as it fails to adequately differentiate between adipose tissue and lean body mass [26,27]. Investigations between the relationship of maternal BMI and leptin have been conducted predominantly in skim HM, which excludes the fat and cellular components of HM [21,24]. Further, leptin concentrations have been shown to be higher in whole HM compared to skim HM [20,23,28].

In contrast, maternal serum concentrations of adiponectin are lower if weight and BMI are higher [29,30]. Results associating maternal BMI and adiponectin concentrations in HM are conflicting [25], with several studies showing no associations [11,31,32] and two studies counter-intuitively reporting a positive association [33,34]. As with leptin, use of BMI as a measure of maternal adiposity may contribute to these conflicting findings. Also, both increasing [11,35] and decreasing [30,33,36] trends in adiponectin concentrations across the lactation period have been reported.

Similar to its effects on appetite hormones, it is postulated that maternal adiposity influences macronutrient concentrations in HM. Again, results are conflicting. Excessive adipose tissue storage has been shown to impair amino acid and monosaccharide metabolism and transport [37,38], yet increased serum amino acid levels were found in mothers with more adipose tissue [39]. In lactating women, higher concentrations of HM protein were associated with lower BMI in one study [40], yet with higher BMI [41] and higher adiposity [42] in others. BMI also was found to associate positively with concentration of HM galactose [41]. There is a possibility that the effect of maternal body composition (BC) only becomes evident in late but not early lactation, when the fat accumulated during pregnancy is depleted [42]. More precise measurements of maternal adiposity across the lactation period are needed to elucidate effects on HM composition.

This study investigated relationships between maternal adiposity and HM leptin, adiponectin, total protein and lactose. Further, it investigated the relationship between percentage fat mass (%FM) and BMI, and the change in maternal adiposity and component concentrations over the first year of lactation.

## 2. Materials and Methods

### 2.1. Study Participants

Fifty-nine predominantly Caucasian, English-speaking, breastfeeding mothers were recruited via the Australian Breastfeeding Association (ABA) and through external networking. Inclusion criteria were: healthy singletons, gestational age ≥37 weeks, fully breastfed at 2 and/or 5 months [43] and maternal intention to breastfeed until 12 months. The exclusion criterion was: maternal smoking. Participants were recruited during their 2nd, 5th, 9th and 12th month of lactation and invited to come back at any subsequent time points. Twenty-one participants contributed samples at two or more time points. All participants provided informed written consent and answered a secure online questionnaire that was administered and securely stored at the university. This study was approved by the Human Research Ethics Committee of The University of Western Australia (RA/4/1/4253) and registered with the Australian New Zealand Clinical Trials Registry (ACTRN12616000368437).

### 2.2. Human Milk Sample Collection

HM samples were collected on site at our research laboratory at King Edward Memorial Hospital for Women (Subiaco, Perth, Australia). Pre-feed and post-feed milk samples (~5 mL each) were obtained from the breast(s) the infant fed from by hand expression or with a breast pump and were analysed separately. Samples were collected between 9:30 and 11:30 a.m. to minimise possible circadian influences on the milk composition. Samples were stored at −20 °C for later biochemical analysis. 

### 2.3. Anthropometry and Body Composition

Maternal weight was measured using an electronic scale (±0.1 kg; Seca, Chino, CA, USA). Height was self-reported by participants or measured against a calibrated marked wall (accuracy ± 0.1 cm). BMI was calculated as kg/m^2^. 

Percentage fat mass (%FM) was measured with whole body bioimpedance (wrist to ankle) using an ImpediMed SFB7 tetra-polar bioelectrical impedance analyser (Impedimed, Brisbane, Australia) with the participant in a supine position on a non-conductive surface according to the manufacturer’s instructions. Before each session, the external calibration of the bioelectrical impendence analyser was tested with a calibration Test Cell (ImpediMed, Brisbane, Australia). Ten consecutive measurements of %FM were taken within 1–2 min and averaged. Within-participant coefficient of variation for maternal %FM was 0.21%. All measurements were made after the breastfeeding session. 

### 2.4. Leptin and Adiponectin Measurements

Leptin concentrations in whole and skim HM were measured using the DuoSet Human Leptin enzyme-linked immunosorbent assay (ELISA) (R&D Systems, Minneapolis, MN, USA) as described previously [28]. The detection limit was 0.05 ng/mL with a recovery of 96.3% ± 1.2% (*n* = 10) for skim milk and 97.1% ± 9.1% (*n* = 10) for whole milk leptin and an inter-assay coefficient of variation (CV) of <7.2%.

Adiponectin was measured in whole HM using the Biovendor Human Adiponectin Sandwich ELISA kit, (Life Technologies, Asheville, NC, USA). The detection limit was 1 ng/mL, with a recovery of 96.2% ± 3.2% (*n* = 10) and an inter-assay CV of <2.5%.

### 2.5. Protein and Lactose Measurements

Protein content was measured using the Bradford assay according to the methods of Mitoulas et al. [44]. The detection limit was 1.03 g/L, with a recovery of 97.2% ± 1.4% (*n* = 10) and an inter-assay CV of <1.9%.

Lactose concentration was measured using the enzymatic–spectrophotometric method outlined by Kuhn et al. [45] according to the methods of Mitoulas et al. [44]. The detection limit was 30 mM, with a recovery of 98.2% ± 4.1% (*n* = 10) and an inter-assay CV of <3.5%.

### 2.6. Statistical Analyses

Statistical analyses were performed using R 2.15.1 for Windows [46]. The packages nlme [47] and lattice [48], and RColorBrewer [49] were used for linear mixed effects modeling and data representation respectively. Descriptive statistics are reported as mean ± standard deviation (SD) and range unless otherwise stated; model parameters are presented as estimate ± standard error (SE).

In order to collect systematic information over time and at fixed moments in time and to make better use of the collected data, a combined data approach that considers individual-level random effects to account for participants measured at two or more study sessions was adopted. We further contrasted the results from the combined data and from the longitudinal subset to confirm our findings. 

During this study, infants were measured at least at one of the four time points (2, 5, 9 and 12 months postpartum). An approximate sample size was calculated using the ‘Linear multiple regression: fixed model: r^2^ increase’ option in G*Power [50] as if this was a cross-sectional study with equal numbers at each time. Allowing four predictors (one main effect, three group contrasts), α = 0.05 and 22 participants at each time point (88 sample points = 22 participants × 4 time points) gave the study power of 0.80 to detect an effect size of 0.15. This approach was selected as there is no closed form expression suitable for the calculation of sample sizes for this research design [51], with the consideration that longitudinal study design is more powerful. To maintain predicted power and to address issues relating to missed visits, such as inability to attend due to illness and unwillingness of mothers approached at 2 months (*n* = 8) to commit to a study that requires breastfeeding to 12 months, the recruitment of participants continued past 22, resulting in 111 sessions for 59 (21 longitudinal, 38 cross-sectional) participants.

BC data at 9 months of lactation is missing for two longitudinal participants. Missing data also occurred for all milk components due to insufficient milk sample volumes. Missing data was dealt with using complete case (regression models) or available case (descriptive statistics) approaches. Milk samples were not pooled for biochemical analysis; thus, measures were not averaged. Sample sizes are presented in Table 1.

Linear models were used to investigate associations between maternal BMI or %FM (predictors) and each of the composition variables (responses), with and without controlling for month of lactation (four-level factor or linear predictor). Associations with month of lactation were assessed using omnibus F-tests and specific post-hoc tests comparing each of the subsequent time points with 2 months. Appropriate random effects were selected by comparing four models for each analysis using a likelihood ratio test. Models were (a) linear regression, and linear mixed effects models with random effects of one of: (b) effect of general inter-individual variation present in the study population; (c) effect of the month of lactation samples were collected at, in addition to inter-individual variation; and (d) the effect of pre- and post-feed samples along with inter-individual variation. Whether the overall effect of maternal adiposity on HM component concentrations differs by month of lactation was also investigated by including interactions between BMI/%FM and the month of lactation (factor only). To allow for realistic interpretation of the intercept values in the model outputs, maternal BMI and %FM have been centred at the upper bounds of the ‘healthy’ range (25 kg/m^2^ for BMI, and 33% for %FM) [52,53]. Where significant outlier values were identified from a kernel density plot, models were run with and without these values to determine how they might be influencing the findings.

An intercept-only linear mixed effects model was used to calculate the coefficient of variation for maternal %FM measurements (*n* = 10, 10 measurements each).

## 3. Results

### 3.1. Participants

Participant adiposity measures and HM components’ concentrations are shown in Table 2. Mean maternal age was 33.4 ± 4.2 years and parity was 1.8 ± 0.8 at the start of the study. At the first session attended at either 2, 5, 9 or 12 months postpartum participants were classified as being underweight (BMI < 18.5, 5%, *n* = 3; %FM < 21, 7%, *n* = 4), of normal weight (BMI 18.5–24.9, 54%, *n* = 32; %FM 21–32.9, 50%, *n* = 29), overweight (BMI 25–29.9, 24%, *n* = 14; %FM 33–38.9, 28%, *n* = 16) or obese (BMI > 30, 17%, *n* = 10; %FM > 39, 15%, *n* = 9) [53]. Infant male/female ratio was 33/26.

### 3.2. Changes in Components’ Concentration with Feeding (Pre- and Post-Feed)

HM component concentrations did not differ between pre-feed and post-feed samples in univariate models or after accounting for the month of lactation as a linear effect model or as a factor (Table 3). 

### 3.3. Associations in Combined Subset

#### 3.3.1. Differences in Concentrations of Human Milk Components at Different Months of Lactation

Table 4 presents the changes in HM components’ concentrations in the combined subset (*n* = 57) at four time points during first 12 months of lactation. 

While component concentrations differed by the month of lactation within participants for all components (lactose: *p* = 0.031; adiponectin, whole and skim milk leptin, protein: *p* < 0.001), no consistent month of lactation-related patterns were seen for whole milk leptin (*p* > 0.47), protein (*p* > 0.37) and lactose (*p* > 0.26). 

Skim milk leptin decreased non-linearly over the months of lactation (univariate: *p* = 0.024). Post-hoc tests showed that adiponectin concentration at 9 months was −2.27 ± 0.88 ng/mL lower (*p* = 0.013) than that at 2 months of lactation (univariate: *p* = 0.042) (Table 4).

#### 3.3.2. Associations between Maternal Adiposity and HM Leptin

Table 4 presents associations between adiposity and HM components’ concentrations seen in the combined subset (*n* = 57) at four time points during first 12 months of lactation.

Associations between Maternal Adiposity and HM Leptin

**Figure 1:**
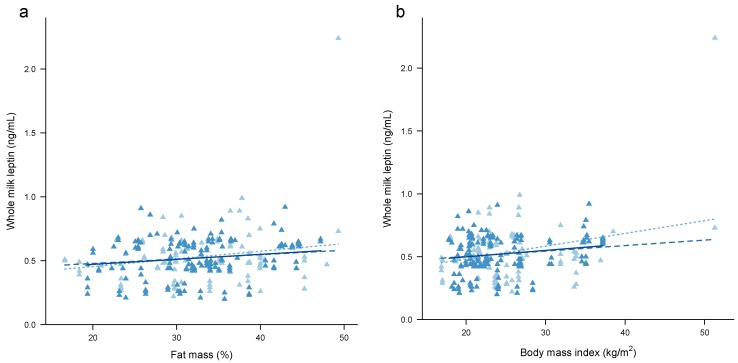
Associations between whole human milk (HM) leptin and (**a**) maternal percentage fat mass; and (**b**) maternal BMI. Combined subset data points (measured in pre- and post-feed samples) are shown as cross-sectional (pale blue) and longitudinal (dark blue). Lines are fixed effects from univariate linear mixed effect models: pale blue dotted line—combined cohort (Table 4); medium blue dashed line—combined cohort with outliers removed (Section 3.3.2); solid dark blue line—longitudinal cohort (Table S1).

**Figure 2:**
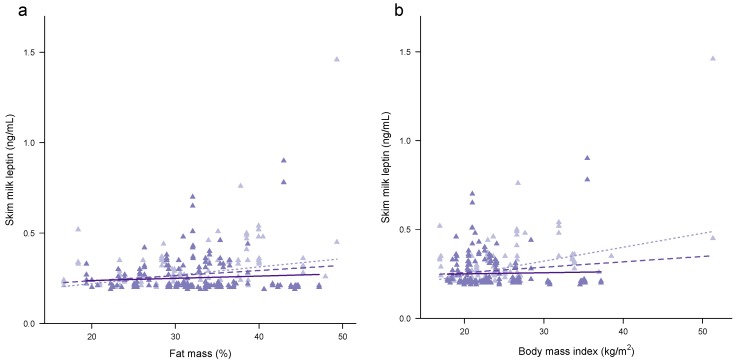
Associations between skim human milk (HM) leptin and (**a**) maternal percentage fat mass; and (**b**) maternal BMI. Combined subset data points are shown as cross-sectional (pale purple) and longitudinal (dark purple). Lines are fixed effects from univariate linear mixed effect models: pale purple dotted line—combined cohort (Table 4); medium purple dashed line—combined cohort with outliers removed (Section 3.3.2); solid dark purple line—longitudinal cohort (Table S1).

Higher %FM and BMI were associated with higher concentrations of both whole (Figure 1a,b) and skim milk (Figure 2a,b) leptin (Table 4). Accounting for the month of lactation as a main linear effect or as a factor did not change the associations with %FM and BMI for both whole and skim milk leptin. Significant negative interactions were seen between %FM and the month of lactation for whole milk leptin, (2 m: reference; 5 m: −0.02 ± 0.01, *p* = 0.023; 9 m: −0.02 ± 0.01, *p* = 0.003; 12 m: −0.03 ± 0.01, *p* < 0.001; month of lactation as a factor: *p* = 0.008), and for skim milk leptin (2 m: reference; 5 m: −0.02 ± 0.01, *p* < 0.001; 9 m: −0.02 ± 0.01, *p* < 0.001; 12 m: −0.02 ± 0.01, *p* = 0.002; month of lactation as a factor: *p* < 0.001); and also between BMI and the month of lactation for whole milk leptin, (2 m: reference; 5 m: −0.02 ± 0.01, *p* = 0.026; 9 m: −0.03 ± 0.01, *p* < 0.001; 12 m: −0.03 ± 0.01, *p* < 0.001; month of lactation as a factor: *p* < 0.001), and for skim milk leptin (2 m: reference; 5 m: −0.02 ± 0.01, *p* < 0.001; 9 m: −0.02 ± 0.01, *p* < 0.001; 12 m: −0.01 ± 0.01, *p* = 0.005; month of lactation as a factor: *p* = 0.001), indicating that the association between adiposity and leptin weakens over the first 12 months of lactation.

Removing statistically significant outliers resulted in either weakening or an absence of the association between either %FM or BMI and whole and skim milk leptin in the univariate models (%FM: 0.004 ± 0.002 ng/mL, *p* = 0.066; 0.003 ± 0.001 ng/mL, *p* = 0.043, respectively; BMI: 0.004 ± 0.002 ng/mL, *p* = 0.065; 0.004 ± 0.002 ng/mL, *p* = 0.066, respectively) and after accounting for the month of lactation as a linear effect (%FM: 0.004 ± 0.002 ng/mL, *p* = 0.039, age: *p* = 0.17; 0.002 ± 0.001 ng/mL, *p* = 0.12, age: *p* = 0.030, respectively; BMI: 0.005 ± 0.002 ng/mL, *p* = 0.053, age: *p* = 0.25; 0.004 ± 0.002 ng/mL, *p* = 0.039, age: *p* = 0.17, respectively) or as a factor (%FM: 0.004 ± 0.002 ng/mL, *p* = 0.044, age: *p* = 0.37; 0.002 ± 0.001 ng/mL, *p* = 0.14, age: *p* = 0.15, respectively; BMI: 0.004 ± 0.002 ng/mL, *p* = 0.072, age: *p* = 0.53; 0.004 ± 0.002 ng/mL, *p* = 0.044, age: *p* = 0.37, respectively). No interaction between either %FM or BMI and the month of lactation as a factor was seen (%FM: whole milk leptin: *p* = 0.37; skim milk leptin: *p* = 0.13; BMI: whole milk leptin: *p =* 0.24; skim milk leptin: *p* = 0.18).

#### 3.3.3. Associations between Maternal Adiposity and HM Adiponectin

HM adiponectin was not significantly associated with either %FM or BMI in the univariate models (Table 4) or after accounting for the month of lactation. No interactions were seen between the month of lactation as a factor and either %FM (*p* = 0.51) or BMI (*p* = 0.62). 

Removing a statistically significant outlier did not change the conclusion (%FM: *p* ≥ 0.50; BMI: *p* ≥ 0.083) and no interaction between the month of lactation and either %FM (*p* = 0.54) or BMI (*p* = 0.081) was seen.

#### 3.3.4. Associations between Maternal Adiposity and HM Protein

**Figure 3:**
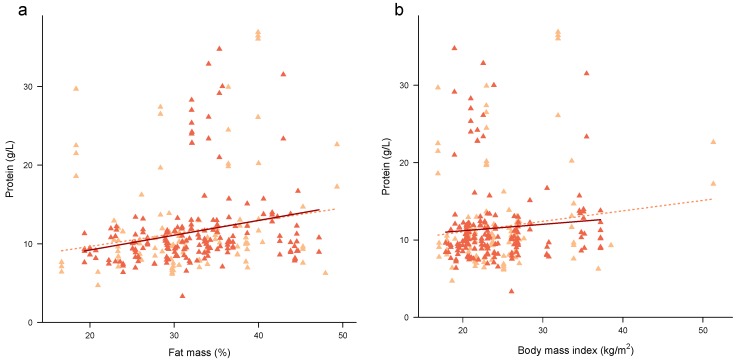
Associations between protein concentration and (**a**) maternal percentage fat mass; and (**b**) maternal BMI. Combined subset data points are shown as cross-sectional (pale orange) and longitudinal (dark orange). Lines are fixed effects from univariate linear mixed effect models: pale orange dotted line—combined cohort (Table 5); solid dark red line—longitudinal cohort (Table S1).

Higher %FM was associated with higher concentrations of protein in HM in univariate model (Table 5; Figure 3a). Accounting for the month of lactation made the association between %FM and protein concentrations weaker but still significant. BMI was not associated with concentrations of protein in HM in the univariate model (Figure 3b) or after accounting for the month of lactation. No interaction with the month of lactation as a factor was seen for both %FM (*p* = 0.21) and BMI (*p* = 0.16).

#### 3.3.5. Associations between Maternal Adiposity and HM Lactose

Neither %FM or BMI were associated with concentrations of lactose in HM in univariate model (Table 5) or after accounting for the month of lactation. No interaction with the month of lactation as a factor was seen for BMI (*p* = 0.19), but a significant positive interaction was seen between %FM and the month of lactation (2 m: reference; 5 m: 0.12 ± 0.33, *p* = 0.71; 9 m: −0.14 ± 0.30, *p* = 0.63; 12 m: 0.58 ± 0.31, *p* = 0.068; month of lactation as a factor: *p* = 0.029), indicating that association between %FM and lactose strengthens over the first 12 months of lactation.

### 3.4. Associations in the Longitudinal Subset

#### 3.4.1. Participants

Longitudinal (*n* = 21) participants’ characteristics and HM components’ concentrations are shown in Table S2. Participants in longitudinal subset were generally leaner than in the combined subset, but none were underweight. At the first session they were classified as: normal weight (BMI 18.5–24.9, 67%, *n* = 14; %FM 21–32.9, 57%, *n* = 12), overweight (BMI 25–29.9, 19%, *n* = 4; %FM 33–38.9, 29%, *n* = 6) or obese (BMI > 30, 14%, *n* = 3; %FM > 39,14%, *n* = 3) [53]. Infant male:female ratio was 10:11.

#### 3.4.2. Longitudinal Changes in Concentrations of Human Milk Components

Table S1 presents the changes in HM component concentrations in the longitudinal subset (*n* = 21 participants, 73 sessions). While component concentrations differed by the month of lactation within participants for all components (lactose: *p* = 0.020; adiponectin, whole and skim milk leptin, protein: *p* < 0.001), no consistent month of lactation-related patterns were seen for adiponectin (*p* > 0.32), whole milk leptin (*p* > 0.11) or lactose (*p* > 0.46). Although the overall pattern for protein was not significant (*p* > 0.10), post-hoc tests showed that protein concentration at 9 months was 3.80 ± 1.65 g/L lower than that at 2 months of lactation (univariate, *p* = 0.027). Skim milk leptin decreased non-linearly over the months of lactation (univariate: *p* = 0.007).

#### 3.4.3. Associations between Maternal Body Mass Index and Percentage Fat Mass

A strong relationship (*p* < 0.001) was observed between maternal BMI and %FM in the longitudinal subset, with a one-unit increase in BMI associated with a 1.07% ± 0.17% increase in %FM. After accounting for the month of lactation there was a significant overall difference in %FM with the month of lactation (*p* = 0.008) and the association between maternal BMI and %FM remained significant (1.01 ± 0.17, *p* < 0.001).

**Figure 4:**
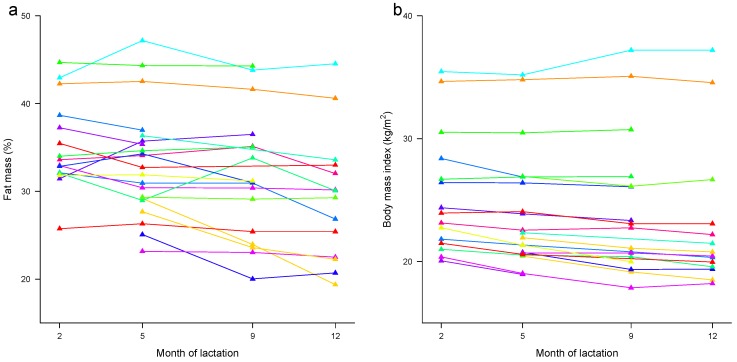
Longitudinal changes in (**a**) maternal percentage fat mass and (**b**) maternal BMI from 2 to 12 months of lactation. Lines are colour-coordinated for the individual participants (e.g., dark orange in panel (**a**) is a same dark orange in panel (**b**)) for illustrative purposes only (*n* = 21).

Over the first year of lactation, maternal %FM decreased in non-linear fashion (largest drop between 9 and 12 months) by more than 2% after accounting for the month of lactation as a factor (−2.26% ± 0.67%, *p* = 0.002; age: *p* < 0.001) or by 0.23% per month after accounting for the month of lactation as a linear effect (−0.23% ± 0.06 %, *p* < 0.001) (Figure 4a).

Over the first year of lactation, maternal BMI decreased in an almost linear fashion; decreasing by −1.05 ± 0.24 kg/m^2^ (*p* < 0.001; month of lactation: *p* < 0.001) over the ten months of the study when accounting for the month of lactation as a factor, or by −0.10 ± 0.02 kg/m^2^ (*p* < 0.001) per month when accounting for the month of lactation as a linear effect (Figure 4b).

#### 3.4.4. Associations between Maternal Adiposity and Human Milk Components in Longitudinal Subset

No associations were seen between measures of maternal adiposity and adiponectin (%FM: *p* > 0.36; BMI: *p* > 0.39), whole (%FM: *p* > 0.051; BMI: *p* > 0.082) and skim milk leptin (%FM: *p* > 0.51; BMI: *p* > 0.78) and lactose concentrations (%FM: *p* > 0.56; BMI: *p* > 0.68) in either univariate models or after accounting for the month of lactation (Table S1).

Higher %FM was associated with higher protein concentration in the univariate model (0.19 ± 0.09 g/L, *p* = 0.035); when the month of lactation was accounted for, this association was no longer seen (0.14 ± 0.09 g/L, *p* = 0.12).

No interaction with the month of lactation as a factor was seen for adiponectin (%FM: *p* = 0.87; BMI: *p* = 0.52), whole milk leptin (%FM: *p* = 0.62; BMI: *p* = 0.36), skim milk leptin (%FM: *p* = 0.37), protein (%FM: *p* = 0.78; BMI: *p* = 0.25) and lactose (%FM: *p* = 0.22; BMI: *p* = 0.30). 

Significant negative interactions were seen between BMI and the month of lactation for skim milk leptin, (2 m: reference; 5 m: −0.02 ± 0.01, *p* = 0.005; 9 m: −0.01 ± 0.01, *p* = 0.059; 12 m: −0.01 ± 0.01, *p* = 0.059; month of lactation as a factor: *p* = 0.043), indicating that association between BMI and skim milk leptin weakens over the first 12 months of lactation.

## 4. Discussion

The hormonal regulation of appetite plays a central role in infant developmental programming facilitating a lifelong healthy balance between energy intake and expenditure [9]. Concentrations of appetite hormones and macronutrients present in HM influence regulation of appetite, energy expenditure pathways and growth trajectory in the developing infant [8,54,55]. Maternal adiposity may play a critical role in regulation of concentrations of HM leptin, adiponectin, protein and lactose, and thus of the ingested dose of these components by the infant. This study found some associations of higher maternal adiposity with higher concentrations of HM leptin and protein, but no associations with adiponectin or lactose. Concentrations of whole milk leptin, adiponectin, protein and lactose did not systematically change with milk removal during a breastfeed, or over the first year of lactation—a period which includes both exclusive breastfeeding and the introduction of complementary foods.

This study has shown that the greater the maternal %FM or BMI, the higher the concentrations of HM leptin. This is logical in that a greater amount of adipose tissue results in more leptin secreted into the circulation and thus increased amounts are transferred into the milk. In young infants, HM is believed to be a major source of leptin, due to immature endogenous leptin-synthesising mechanisms [56]. Leptin may provide both short and long term appetite control acting as a neurotrophic molecule targeting the hypothalamus to stimulate neural connections with other key appetite nuclei [57]. Higher HM leptin concentrations are associated with lower infant weight, weight gain and adiposity [8,58] while higher concentrations in infant serum are associated with greater lean body mass (total body water) [59,60], suggesting a pivotal role in regulating infant growth and BC. 

One might speculate that increased amounts of HM leptin supplied to an infant may be detrimental to the development of infant appetite control and growth. However, concentrations of leptin do not reflect the 24-h dose received by the infant and indeed 24-h milk intakes are variable between dyads [61]. In particular, obese mothers are more likely to have reduced milk production due to incomplete differentiation of mammary epithelial cells [62]. Conversely, lean mothers with very low plasma leptin concentrations may produce milk with low leptin levels, thus signalling marginal environment and promoting rapid infant growth while supported by maternal metabolism [63]. Lower infant serum leptin levels would thus reduce the neurotrophic effects on the hypothalamic appetite circuitry and lower satiety stimulation [8,13]. Accordingly, maintenance of healthy maternal adiposity during pregnancy and lactation may ensure appropriate levels of leptin supply to the infant, supporting the optimal programming of appetite control in infancy.

Whilst the lack of relationship between maternal %FM/BMI and HM leptin in the longitudinal subset (Figure 1 and Figure 2) may seem counter-intuitive, there is a marked reduction in the variability of both the maternal BC and HM leptin levels, restricting applicability of the results (Tables S1 and S2). However, HM leptin concentrations have not been shown to differ between obese and non-obese mothers [64]. Furthermore, the contribution by the lactocytes to leptin levels in HM [19,20] is not known. These limitations may explain why some studies (*n* = 11) find an association and others do not (*n* = 4) [25]. Moreover, the majority of previous studies analysed leptin in skim HM in cross-sectional cohorts and are restricted to the first and third months postpartum [25], limiting their ability to determine leptin profiles during lactation in women of varying BC. Only three studies have analysed %FM using dual-energy X-ray absorptiometry (DXA) [24], skinfold measures [63] and bioelectrical impedance analysis [65] and they found a strong association between %FM and skim milk leptin, consistent with the results from our study. Interestingly, Khodabakhshi et al. (2015) [65] found association between HM leptin and both %FM and BMI only in the subset of mothers of obese infants but not in the subset of mothers of normal infants, although these two groups did not differ by BMI or leptin concentration.

Concentrations of adiponectin in HM were not related to maternal %FM and BMI in either the combined or longitudinal subsets in this study. This is not consistent with the physiological inverse relationship between the number of white adipocytes and serum adiponectin levels in humans [29], however it is in agreement with a recent meta-analysis by Andreas et al. [25] who reported the absence of a consistent relationship, either positive or negative, between maternal BMI and colostrum or skim HM adiponectin. We have now expanded the absence of a relationship to maternal %FM and whole HM adiponectin. Thus, it is unlikely that maternal adiposity plays a major role in influencing HM adiponectin levels and it suggests the majority of HM adiponectin may be synthesized and controlled by the mammary gland [66] highlighting the importance of this HM hormone for the infant. Indeed, it appears that HM adiponectin levels regulate infant growth with higher levels of HM adiponectin being associated with lower weight for age (WAZ) and weight for length (WLZ) *z*-scores at 6 months of age [34] and higher WAZ and WLZ scores over the first 2 years of life [54]. These results were also supported by Brunner et al. [32], who found that higher concentrations of HM adiponectin at 6 weeks were associated with lower infant fat-free mass and weight at 4 months as well as greater weight and fat mass at 1 and 2 years of age. The follow up at 3, 4 and 5 years of age has not shown any relationship with the exception of the positive association between HM adiponectin levels at 4 months postpartum and fat mass at 4 years [67]. This reversal of the initial trend in early life is speculated to be related to the timing of cessation of breastfeeding [54]. High HM adiponectin levels may initially down-regulate infant growth, and later promote adipogenesis and adipocyte hypertrophy [68]. Conversely lean populations with lower concentrations of HM adiponectin demonstrate a positive association with the infant WAZ scores. This suggests that the association between HM adiponectin and infant growth may in fact be parabolic, further highlighting the pleiotropic effects of adiponectin during development and the adaptive mechanisms that humans display in the marginal environments [66]. Our recent study of gastric emptying and breastfeeding patterns in fully breastfed term infants has established that higher concentrations and doses of HM adiponectin are associated with longer times between breastfeeds (gastric emptying time) [69], which may partially explain the growth-regulating effect of adiponectin in some populations. As such, investigations into other factors that may affect adiponectin concentrations in HM and its effect on infant growth and BC development are warranted.

The few studies investigating relationships between HM protein composition and maternal adiposity are contradictory, with some reporting a positive association between protein and maternal adiposity (BMI or percentage ideal weight) [41,42] and one a negative association between protein and BMI [40]. Increased serum amino acid concentrations are present in mothers with more adipose tissue [39], leading to more amino acids transferred to the breast and HM [70], explaining the positive relationship between maternal adiposity and HM protein concentrations [42]. This study has found that higher %FM but not BMI was associated with higher protein concentrations, which is similar to Quinn et al. [63] who reported %FM to be more precise measure of adiposity reporting stronger correlation with HM leptin, thus the more precise measure of maternal BC is desirable in mechanistic research.

The measured concentrations of lactose in our cohort were consistent with the normal range in HM [71] and were not related to maternal adiposity profiles. HM provides a constant source of carbohydrates to the infant during early life, ensuring adequate nourishment, maturation and development of their relatively immature physiological systems [41,72,73]. Given the fact that lactose is important for maintaining a constant osmotic pressure in HM [74], maternal adiposity is not expected to have a significant impact on lactose concentration. 

Despite significant changes in maternal %FM and BMI over the 12 months of lactation, and the introduction of complimentary foods, the measured HM components have remained relatively stable (Table 4, Table 5 and Table S2). However, adiponectin concentration decreased significantly in the combined subset as well as in the concentration of protein in the longitudinal subset, both at 9 months only. The temporary drop in protein concentration was consistent with differences described by Nommsen et al. [42]. Whereas some studies have reported a decrease in concentrations of leptin [75] and adiponectin [30,33,36,66] (measured predominantly in skim HM), others found the opposite trend for adiponectin [11,35], no change [76] or significant fluctuations [11,21] for leptin. More research is required to clarify these relationships.

In this study we have measured leptin in both skim and whole milk. Interestingly, longitudinal changes observed in concentrations of skim milk leptin in this study were not confirmed in whole HM. Caution in the interpretation of leptin concentrations measured in skim HM should be exercised, as whole milk measures are more indicative of the level of hormone consumed by the infant [28]. The consistent concentrations of adiponectin and leptin in whole HM over the first 12 months of life may be indicative of a continuing roles of appetite programming, priming neural connections involved in the appetite circuitry, thereby contributing to long-lasting enhanced appetite control and BC of breastfed infants throughout life. 

Sampling protocols are of prime importance when investigating relationships between HM components and BC. In this study we confirmed no systematic change between pre- and post-feed samples for concentrations of protein, lactose and whole and skim HM leptin throughout the first 12 months of lactation (Table 3), and report for the first time that whole milk adiponectin concentrations also do not differ significantly pre- to post-feed. Care however should still be taken as fat content [77], ghrelin [75] and glucagon-like peptide 1 (GLP-1) [24] change between pre- and post-feed, further highlighting the importance of prudent sampling.

The limitations of this study are the modest number of longitudinal participants, resulting from time constraints associated with multiple measurement time points. Further, our population was predominantly Caucasian and of high social-economic status; therefore, the results may not be applicable to participants from other backgrounds.

## 5. Conclusions

This study found that elevated maternal adiposity was associated with higher concentrations of leptin and protein of HM in a cross-sectional cohort however; these findings were not confirmed in a smaller longitudinal cohort. Clarification of the relationships between maternal body composition and human milk appetite regulators will identify periods of lactation where interventions may influence programming of early appetite control and body composition of breastfed infants.

## Figures and Tables

**Table 1:** Sample sizes used in statistical analyses.

Month of Lactation	2	5	9	12	Total
Participants *					
Cross-sectional	-	-	-	-	38
Longitudinal	-	-	-	-	21
Total	-	-	-	-	59
Sessions					
Cross-sectional	8	8	13	9	38
Longitudinal	15	21	19	18	73
Total	23	29	32	27	111
Samples (complete cases)					
Cross-sectional	19	21	33	27	100
Longitudinal	41	55	47	40	183
Total	60	76	80	67	283
Samples (available cases) **					
Whole milk adiponectin	66	79	86	72	303
Whole milk leptin	66	79	86	72	303
Skim milk leptin	62	77	85	71	295
Total protein	64	78	87	69	298
Lactose	65	78	86	67	296

* The number of participants at each time point is the same as the number of sessions, thus not specified in the table; ** The number of samples (including pre-feed and post-feed) analysed; this differs by component.

**Table 2:** Maternal adiposity and human milk components concentrations presented at the months after birth for combined subset (*n* = 59) expressed as mean ± standard deviation (SD, range). Some participants (*n* = 21) contributed milk samples at multiple time points.

Month of Lactation	2	5	9	12	Total
	(*n* = 23)	(*n* = 29)	(*n* = 32)	(*n* = 27)	(*n* = 111)
Maternal BMI ^a^	27.0 ± 7.3	23.5 ± 4.5	23.9 ± 5.2	24.4 ± 5.5	24.6 ± 5.7
	(20.1–51.3)	(17.0–35.2)	(16.9–37.2)	(18.2–37.2)	(16.9–51.3)
Maternal fat mass (%)	34.9 ± 6.4	32.5 ± 6.0	30.9 ± 7.9	31.3 ± 7.2	32.3 ± 7.0
	(19.6–49.3)	(23.2–47.2)	(16.7–47.9)	(19.4–45.3)	(16.7–49.3)
Whole milk leptin (ng/mL)	0.55 ± 0.29	0.50 ± 0.17	0.53 ± 0.15	0.54 ± 0.13	0.53 ± 0.19
	(0.21–2.24)	(0.20–0.85)	(0.21–0.99)	(0.24–0.89)	(0.20–2.24)
Skim milk leptin (ng/mL)	0.34 ± 0.21	0.27 ± 0.07	0.26 ± 0.09	0.26 ± 0.08	0.28 ± 0.12
	(0.19–1.46)	(0.20–0.48)	(0.19–0.76)	(0.19–0.54)	(0.19–1.46)
Adiponectin (ng/mL)	11.12 ± 4.39	9.30 ± 3.94	8.46 ± 2.26	11.07 ± 7.88	9.88 ± 5.05
	(5.62–25.62)	(5.17–29.67)	(4.56–20.29)	(4.74–54.92)	(4.56–54.92)
Total protein (g/L)	12.94 ± 6.15	11.7 ± 5.70	10.83 ± 4.63	12.83 ± 6.74	11.96 ± 5.82
	(6.54–31.51)	(7.00–34.76)	(3.32–29.69)	(6.60–36.89)	(3.32–36.89)
Lactose (g/L)	67.54 ± 9.05	68.07 ± 8.10	68.37 ± 8.83	69.70 ± 9.11	68.41 ± 8.75
	(50.35–89.06)	(50.92–110.07)	(51.81–100.05)	(51.00–98.36)	(50.35–110.07)

Data are mean ± SD and ranges. Concentrations of components are measured in both pre- and post-feed milk samples. ^a^ BMI—body mass index.

**Table 3:** Relative concentrations of pre-feed human milk samples compared to post-feed samples with and without accounting for possible month of lactation effects.

Predictor	Univariate ^a^		Accounting for Month of Lactation (Linear) ^b^		Accounting for Month of Lactation (Factor) ^b^	
	PE ± SE	*p*	PE ± SE	*p*	PE ± SE	*p*
Adiponectin (ng/mL)	0.45 ± 0.42	0.29	0.45 ± 0.42	0.30	0.45 ± 0.42	0.29 ^c^
Leptin (ng/mL)						
*Whole milk*	−0.008 ± 0.016	0.65	−0.008 ± 0.017	0.65	0.007 ± 0.016	0.66
*Skim milk*	−0.002 ± 0.009	0.84	−0.002 ± 0.009	0.86 ^c^	−0.001 ± 0.009	0.87 ^c^
Protein (g/L)	−0.14 ± 0.32	0.68	−0.14 ± 0.32	0.68	−0.14 ± 0.32	0.68
Lactose (g/L)	−0.36 ± 0.91	0.69	−0.38 ± 0.88	0.66	−0.39 ± 0.88	0.66

Data are parameter estimate ± SE. Analyses were run on pre- and post-feed samples using complete case approach. ^a^ Effects of predictors taken from univariate linear mixed effects models; ^b^ Effects of predictors taken from linear mixed effects models that accounted for the month of lactation as linear main effect or as a factor; ^c^ Month of lactation is significant (*p* < 0.036). PE—parameter estimate; SE—standard error.

**Table 4:** Associations between human milk appetite hormones and maternal adiposity. Values are parameter estimates ± standard error (*n* = 57). Some participants (*n* = 21) contributed milk samples at multiple time points.

Predictor	Adiponectin (ng/mL)		Whole Milk Leptin (ng/mL)		Skim Milk Leptin (ng/mL)	
	PE ± SE	*p*	PE ± SE	*p*	PE ± SE	*p*
*Univariate models* ^b^						
BMI	0.10 ± 0.07	0.17	0.01 ± 0.003	**<0.001**	0.008 ± 0.002	**<0.001** ^a^
%FM	0.01 ± 0.06	0.81	0.006 ± 0.002	**0.008**	0.005 ± 0.002	**0.007**
Month ^d^	-	**0.042**	-	0.52	-	**0.024**
Intercept	10.58 ± 0.71	-	0.55 ± 0.03	-	0.34 ± 0.02	-
5 ^e^	−1.39 ± 0.87	0.12	−0.06 ± 0.05	0.18	−0.07 ± 0.03	**0.038**
9 ^e^	−2.27 ± 0.88	**0.013**	−0.01 ± 0.05	0.75	−0.08 ± 0.03	**0.011**
12 ^e^	−0.26 ± 0.93	0.78	−0.02 ± 0.05	0.72	−0.10 ± 0.03	**0.005**
*Adjusted model for %FM (month of lactation as linear main effect)* ^c^						
Intercept	9.76 ± 0.61	-	0.52 ± 0.03	-	0.32 ± 0.02	-
%FM	0.008 ± 0.06	0.90	0.006 ± 0.002	**0.007**	0.004 ± 0.002	**0.021**
Month ^d^	−0.05 ± 0.09	0.57	0.003 ± 0.004	0.47	−0.008 ± 0.003	**0.012**
*Adjusted model for %FM (month of lactation as a factor)* ^c^						
Intercept	10.59 ± 0.72	-	0.54 ± 0.03	-	0.34 ± 0.02	-
%FM	−0.01 ± 0.06	0.86	0.006 ± 0.002	**0.009**	0.004 ± 0.002	**0.028**
Month ^d^	-	**0.044**	-	0.51	-	0.063
5 ^e^	−1.40 ± 0.88	0.12	−0.04 ± 0.04	0.34	−0.06 ± 0.03	0.070
9 ^e^	−2.30 ± 0.90	**0.014**	0.01 ± 0.05	0.76	−0.07 ± 0.03	**0.031**
12 ^e^	−0.29 ± 0.95	0.77	0.007 ± 0.05	0.88	−0.09 ± 0.03	**0.011**
*Adjusted model for BMI (month of lactation as linear main effect)* ^c^						
Intercept	9.76 ± 0.60	-	0.52 ± 0.03	-	0.33 ± 0.02	-
BMI	0.09 ± 0.07	0.19	0.01 ± 0.003	**<0.001**	0.008 ± 0.002	**<0.001**
Month ^d^	−0.04 ± 0.09	0.62	0.002 ± 0.004	0.59	−0.008 ± 0.003	0.005
*Adjusted model for BMI (month of lactation as a factor)* ^c^						
Intercept	10.51 ± 0.71	-	0.54 ± 0.003	-	0.34 ± 0.02	-
BMI	0.07 ± 0.07	0.33	0.01 ± 0.003	**<0.001**	0.007 ± 0.002	**0.001**
Month ^d^	-	0.063	-	0.74	-	**0.039**
5 ^e^	−1.23 ± 0.89	0.17	−0.03 ± 0.04	0.50	−0.05 ± 0.03	0.13
9 ^e^	−2.13 ± 0.89	**0.021**	0.01 ± 0.04	0.78	−0.07 ± 0.03	**0.025**
12 ^e^	−0.16 ± 0.94	0.87	0.004 ± 0.05	0.92	−0.09 ± 0.03	**0.007**

Data are parameter estimate ± SE. Analyses were run on pre- and post-feed samples using complete case approach. ^a^ Significant *p*-values are in bold font; ^b^ Effects of predictors taken from univariate linear mixed effects models; ^c^ Effects of predictors taken from linear mixed effects models that accounted for the month of lactation as linear main effect or as a factor; ^d^ Omnibus F-test; ^e^ Post-hoc test with reference 2 months. Abbreviations: BMI—body mass index; %FM—percentage fat mass; PE—parameter estimate; SE—standard error.

**Table 5:** Associations between human milk macronutrients and maternal adiposity. Values are parameter estimates ± standard error (*n* = 57). Some participants (*n* = 21) contributed milk samples at multiple time points.

Predictor	Lactose (g/L)		Protein (g/L)	
	PE ± SE	*p*	PE ± SE	*p*
*Univariate models* ^b^				
BMI	0.06 ± 0.14	0.66	0.14 ± 0.09	0.14
%FM	0.08 ± 0.11	0.48	0.16 ± 0.07	**0.028** ^a^
Month ^d^	-	0.65	-	0.37
Intercept	67.32 ± 1.43	-	12.90 ± 1.11	-
5 ^e^	0.56 ± 1.89	0.77	−1.39 ± 1.48	0.35
9 ^e^	1.31 ± 1.88	0.49	−2.47 ± 1.47	0.10
12 ^e^	2.35 ± 1.97	0.24	−0.63 ± 1.55	0.69
*Adjusted model for %FM (month of lactation as linear main effect)* ^c^				
Intercept	68.89 ± 1.13	-	11.93 ± 0.87	-
%FM	0.09 ± 0.11	0.43	0.16 ± 0.07	**0.036**
Month ^d^	0.08 ± 0.15	0.59	−0.03 ± 0.14	0.84
*Adjusted model for %FM (month of lactation as a factor)* ^c^				
Intercept	67.58 ± 1.44	-	12.54 ± 1.11	-
%FM	0.06 ± 0.11	0.55	0.15 ± 0.07	0.054
Month ^d^	-	0.67	-	0.56
5 ^e^	0.60 ± 1.85	0.75	−0.94 ± 1.47	0.53
9 ^e^	1.37 ± 1.87	0.50	−1.78 ± 1.49	0.24
12 ^e^	2.27 ± 1.96	0.25	−0.02 ± 1.56	0.99
*Adjusted model for BMI (month of lactation as linear main effect)* ^c^				
Intercept	67.71 ± 2.92	-	12.10 ± 0.88	-
BMI	0.06 ± 0.13	0.63	0.13 ± 0.09	0.16
Month ^d^	0.20 ± 0.17	0.26	−0.07 ± 0.14	0.61
*Adjusted model for BMI (month of lactation as a factor)* ^c^				
Intercept	67.55 ± 1.44	-	12.69 ± 1.12	-
BMI	0.06 ± 0.13	0.66	0.12 ± 0.09	0.21
Month ^d^	-	0.69	-	0.48
5 ^e^	0.63 ± 1.88	0.74	−1.01 ± 1.50	0.50
9 ^e^	1.29 ± 1.87	0.49	−2.14 ± 1.49	0.16
12 ^e^	2.22 ± 1.95	0.26	−0.36 ± 1.56	0.82

Data are parameter estimate ± SE. Analyses were run on pre- and post-feed samples using complete case approach. ^a^ Significant *p*-values are in bold font; ^b^ Effects of predictors taken from univariate linear mixed effects models; ^c^ Effects of predictors taken from linear mixed effects models that accounted for the month of lactation as linear main effect or as a factor; ^d^ Omnibus F-test; ^e^ Post-hoc test with reference 2 months. Abbreviations: BMI—body mass index; %FM—percentage fat mass; PE—parameter estimate; SE—standard error.

## Supplementary Materials

The following are available online at http://www.mdpi.com/2072-6643/9/3/252/s1, Table S1: Longitudinal changes and associations between human milk components and maternal adiposity. Values are parameter estimates ± standard error (*n* = 21), Table S2: Maternal adiposity and human milk components concentrations presented at the months after birth for longitudinal subset (*n* = 21 participants, 73 sessions). Values are mean ± standard deviation (range).
