# Supplementary material for: Associations between Maternal Body Composition and Appetite Hormones and Macronutrients in Human Milk
Source: Nutrients. 2017 Mar 9;9(3):252. doi: 10.3390/nu9030252 (PMC5372915; doi:10.3390/nu9030252)
Supplement: Supplementary file 1 [file nutrients-09-00252-s001.docx]

Supplementary Materials: Associations between Maternal Body Composition and Appetite Hormones and Macronutrients in Human Milk

Sambavi Kugananthan, Zoya Gridneva, Ching T. Lai, Anna R. Hepworth,
Peter J. Mark, Foteini Kakulas and Donna T. Geddes

**Table S1.** Longitudinal changes and associations between human milk components and maternal adiposity. Values are parameter estimates ± standard error (*n* = 21).

| **Predictor** | **Adiponectin**  **(ng/mL)** | | **Whole Milk Leptin**  **(ng/mL)** | | **Skim Milk Leptin**  **(ng/mL)** | | **Lactose (g/L)** | | **Protein (g/L)** | |
| --- | --- | --- | --- | --- | --- | --- | --- | --- | --- | --- |
|  | **PE ± SE** | ***p*** | **PE ± SE** | ***p*** | **PE ± SE** | ***p*** | **PE ± SE** | ***p*** | **PE ± SE** | ***p*** |
| *Univariate models ^b^* | | | | | | | | | | |
| BMI | 0.11 ± 0.13 | 0.41 | 0.005 ± 0.003 | 0.11 | 0.001 ± 0.002 | 0.78 | -0.03 ± 0.11 | 0.78 | 0.08 ± 0.12 | 0.48 |
| %FM | -0.08 ± 0.10 | 0.40 | 0.004 ± 0.003 | 0.12 | 0.001 ± 0.002 | 0.51 | -0.03 ± 0.08 | 0.73 | 0.19 ± 0.09 | **0.035** ^a^ |
| Month ^d^ | - | 0.32 | - | 0.39 | - | **0.007** | - | 0.54 | - | 0.10 |
| Intercept | 10.25 ± 0.99 | - | 0.51 ± 0.04 | - | 0.32 ± 0.03 | - | 68.24 ± 1.15 | - | 13.70 ± 1.21 | - |
| 5 ^e^ | -0.69 ± 1.12 | 0.54 | -0.02 ± 0.05 | 0.67 | -0.05 ± 0.03 | 0.10 | -1.98 ± 1.52 | 0.20 | -1.54 ± 1.58 | 0.34 |
| 9 ^e^ | -1.69 ± 1.17 | 0.15 | 0.06 ± 0.05 | 0.26 | -0.10 ± 0.03 | **0.003** | -1.85 ± 1.58 | 0.24 | -3.80 ± 1.65 | **0.027** |
| 12 ^e^ | 0.32 ± 1.25 | 0.80 | 0.02 ± 0.05 | 0.68 | -0.11 ± 0.03 | **0.003** | -1.92 ± 1.64 | 0.24 | -3.37 ± 1.74 | 0.059 |
| *Adjusted model for %FM (month of lactation as linear main effect) ^c^* | | | | | | | | | | |
| Intercept | 9.84 ± 0.86 | - | 0.49 ± 0.03 | - | 0.31 ± 0.02 | - | 67.23 ± 1.04 | - | 13.08 ± 0.95 | - |
| %FM | -0.09 ± 0.10 | 0.36 | 0.005 ± 0.003 | 0.051 | -0.001 ± 0.002 | 0.67 | -0.05 ± 0.10 | 0.58 | 0.14 ± 0.09 | 0.12 |
| Month ^d^ | -0.05 ± 0.12 | 0.70 | 0.008 ± 0.005 | 0.11 | -0.01 ± 0.003 | **0.001** | -0.17 ± 0.15 | 0.46 | -0.30 ± 0.16 | 0.070 |
| *Adjusted model for %FM (month of lactation as a factor) ^c^* | | | | | | | | | | |
| Intercept | 10.29 ± 1.00 | - | 0.50 ± 0.04 | - | 0.32 ± 0.02 | - | 68.30 ± 1.16 | - | 13.41 ±1.21 | - |
| %FM | -0.07 ± 0.10 | 0.47 | 0.005 ± 0.003 | 0.061 | -0.001 ± 0.002 | 0.71 | -0.05 ± 0.09 | 0.56 | 0.14 ± 0.09 | 0.12 |
| Month ^d^ | - | 0.35 | - | 0.24 | - | **0.008** | - | 0.50 | - | 0.23 |
| 5 ^e^ | -0.71 ± 1.11 | 0.53 | -0.01± 0.05 | 0.81 | -0.05 ± 0.03 | 0.097 | -2.05 ± 1.53 | 0.18 | -1.26 ± 1.57 | 0.43 |
| 9 ^e^ | -1.79 ± 1.17 | 0.13 | 0.07 ± 0.05 | 0.15 | -0.10 ± 0.03 | **0.003** | -1.94 ± 1.59 | 0.23 | -3.33 ± 1.66 | 0.051 |
| 12 ^e^ | 0.07 ± 1.29 | 0.96 | 0.05 ± 0.05 | 0.34 | -0.11 ± 0.04 | **0.003** | -2.16 ± 1.69 | 0.20 | -2.54 ± 1.79 | 0.16 |
| *Adjusted model for BMI (month of lactation as linear main effect) ^c^* | | | | | | | | | | |
| Intercept | 9.78 ± 0.85 | - | 0.50 ± 0.03 | - | 0.31 ± 0.02 | - | 67.14 ± 1.03 | - | 13.33 ± 0.96 | - |
| BMI | 0.11 ± 0.13 | 0.42 | 0.006 ± 0.003 | 0.082 | -0.001 ± 0.002 | 0.82 | -0.05 ± 0.12 | 0.69 | 0.05 ± 0.12 | 0.69 |
| Month ^d^ | -0.002 ± 0.12 | 0.99 | 0.006 ± 0.005 | 0.19 | -0.01 ± 0.003 | **0.001** | -0.11 ± 0.17 | 0.51 | -0.37 ± 0.16 | **0.025** |
| *Adjusted model for BMI (month of lactation as a factor) ^c^* | | | | | | | | | | |
| Intercept | 10.27 ± 0.99 | - | 0.51 ± 0.03 | - | 0.32 ± 0.02 | - | 68.25 ± 1.16 | - | 13.67 ± 1.22 | - |
| BMI | 0.11 ± 0.13 | 0.39 | 0.005 ± 0.003 | 0.10 | -0.001 ± 0.002 | 0.84 | -0.04 ± 0.11 | 0.68 | 0.05 ± 0.11 | 0.65 |
| Month ^d^ | - | 0.32 | - | 0.35 | - | **0.008** | - | 0.53 | - | 0.12 |
| 5 ^e^ | -0.60 ± 1.12 | 0.60 | -0.01 ± 0.05 | 0.78 | -0.05 ± 0.03 | 0.10 | -2.03 ± 1.53 | 0.19 | -1.46 ± 1.59 | 0.37 |
| 9 ^e^ | -1.61 ± 1.17 | 0.18 | 0.06 ± 0.05 | 0.21 | -0.10 ± 0.03 | **0.003** | -1.85 ± 1.58 | 0.24 | -3.73 ± 1.67 | **0.031** |
| 12 ^e^ | 0.49 ± 1.27 | 0.70 | 0.03 ± 0.05 | 0.51 | -0.11 ± 0.03 | **0.003** | -2.01 ± 1.66 | 0.23 | -3.24 ± 1.76 | 0.073 |

Data are parameter estimate ± SE. Analyses were run on pre- and post-feed samples using complete case approach. ^a^ Significant *p* – values are in bold font. ^b^ Effects of predictors taken from univariate linear mixed effects models. ^c^ Effects of predictors taken from linear mixed effects models that accounted for the month of lactation as linear main effect or as a factor. ^d^ Omnibus F-test. ^e^ Post-hoc test with reference 2 months. Abbreviations: BMI – body mass index; %FM – percentage fat mass; PE – parameter estimate; SE – standard error.

**Table S2.** Maternal adiposity and human milk components concentrations presented at the months after birth for longitudinal subset (*n* = 21 participants, 73 sessions). Values are mean ± standard deviation (range).

| **Month of**  **Lactation** | **2**  **(*n* = 15)** | **5**  **(*n* = 21)** | **9**  **(*n* = 19)** | **12**  **(*n* = 18)** | **Total**  **(*n* = 73)** |
| --- | --- | --- | --- | --- | --- |
| Maternal BMI ^a^ | 26.0 ± 5.4  (20.1–35.5) | 23.7 ± 5.0  (19.0–35.2) | 25.9 ± 5.8  (19.2–37.2) | 23.1 ± 6.1  (18.5–37.2) | 24.5 ± 5.5  (18.5–37.2) |
| Maternal fat mass (%) | 35.0 ± 5.3  (25.7–43.0) | 33.0 ± 6.2  (23.2–47.2) | 33.3 ± 7.4  (23.0–44.3) | 29.7 ± 7.6  (19.4–44.5) | 32.7 ± 6.8  (19.4–47.2) |
| Whole milk leptin (ng/mL) | 0.50 ± 0.16  (0.21–0.92) | 0.49 ± 0.17  (0.20–0.82) | 0.57 ± 0.12  (0.21–0.79) | 0.54 ± 0.13  (0.24–0.86) | 0.52 ± 0.15  (0.20–0.92) |
| Skim milk leptin (ng/mL) | 0.32 ± 0.17  (0.19–0.90) | 0.27 ± 0.08  (0.20–0.48) | 0.22 ± 0.03  (0.19–0.35) | 0.23 ± 0.05  (0.19–0.41) | 0.26 ± 0.10  (0.19–0.90) |
| Adiponectin (ng/mL) | 10.75 ± 4.99  (5.62–25.62) | 9.80 ± 4.27  (6.02–29.67) | 8.91 ± 2.60  (6.07–20.29) | 11.02 ± 7.25  (4.74–52.00) | 10.07 ± 4.99  (4.74–52.00) |
| Total protein (g/L) | 13.63 ± 6.80  (6.54–31.51) | 12.42 ± 6.39  (7.09–34.76) | 10.12 ± 2.42  (3.32–15.73) | 10.67 ± 2.44  (6.95–16.80) | 11.71 ± 5.18  (3.32–34.76) |
| Lactose (g/L) | 68.24 ± 10.47  (50.35–89.06) | 66.28 ± 5.84  (50.92–79.48) | 66.40 ± 6.62  (54.74–90.61) | 66.31 ± 6.10  (51.00–79.33) | 66.75 ± 7.33  (50.35–90.61) |

Data are mean ± SD and ranges. Concentrations of components are measured in both pre- and post-feed milk samples. ^a^ BMI - body mass index.
